# Dataset on the microstructure morphology and elemental composition of the palm kernel shell and the coconut kernel shell by TEM/SEM and EDXA/EDS

**DOI:** 10.1016/j.dib.2018.12.024

**Published:** 2018-12-12

**Authors:** Richard Ntenga, Etienne Mfoumou, Alexis Béakou, Martin Tango, Jordan Kamga, Ali Ahmed

**Affiliations:** a Laboratoire d’Analyse, Simulations et Essais (LASE), IUT, Université de Ngaoundéré, Cameroon; b Laboratoire de Mécanique et Productique (LMP), UFD SI, Université de Douala, P.O. Box 27, Douala, Cameroon; c Applied Research & Innovation, Nova Scotia Community College, Dartmouth, Nova Scotia, Canada; d Institut Pascal UMR 6602 - UCA - CNRS - SIGMA, Clermont-Ferrand, France; e Ivan Curry School of Engineering, Acadia University, Wolfville, Nova Scotia, Canada

**Keywords:** Palm kernel shell, Coconut kernel shell, SEM and TEM, EDS and EDXA

## Abstract

Coconut kernel shell (CKS) and palm kernel shell (PKS) powders were analyzed using Surface Electron Microcopy (SEM) and Transmission Electron Microscopy (TEM). The SEM and TEM were combined with Energy Dispersive X-ray Spectroscopy Analysis for elemental composition of the sample materials. The micrographs of all samples were thoroughly examined and explained. The dataset presented herein helps to elucidate the ultrastructure and could suggest expanding traditional applications of the PKS/CKS to novel ones. The data are related to the research article “Insight on the Ultrastructure, Physicochemical and Thermal Characteristics and Applications of the Palm Kernel Shells” (Ntenga et al., 2018).

**Specifications table**

**Table:** 

**Subject area**	Material Science, Physics, Chemistry,
**More specific subject area**	Vegetal Material characterization
**Type of data**	Images (SEM, TEM, X-ray, text files)
**How data were acquired**	Microscopes: SEM and TEM; A JEOL JSM-6900 Low Vacuum SEM with Energy Dispersive Spectroscopy (EDS); A JEOL TEM, with a JSX-1000S Fluorescence Spectrometer X-ray analyzer.
**Data format**	Raw, filtered and analyzed.
**Experimental factors**	TEM samples were sonicated for 5 minutes in distilled water then dropped onto a lacey carbon coated grid. For SEM, the samples were coated with a mixture of gold and palladium.
**Experimental features**	EDS was carried out by using the Integrated Microanalyzer for Images and X-rays (IMIX) system. Coating of samples by a sputter coater (Polaron SC 7640). TEM/EDXA capable of identifying the elements in areas less than 0.5 µm in diameter from carbon to uranium. A Low-Voltage Electron Microscope (LVEM) (between 5–25 kV), thus no need to stain.
**Data source location**	Mbambou (SOCAPALM plantations), Cameroon, 3° 28′ North, 9° 31′ East.
**Data accessibility**	Part of the data are within this article. Supplementary data related to this article can also be found at: http://dx.doi.org/10.17632/f2zcvxgf24.1
**Related research article**	R. Ntenga, E. Mfoumou, A. Béakou, M. Tango, J. Kamga, A. Ahmed, Insight on the Ultrastructure, Physicochemical and Thermal Characteristics and Applications of the Palm Kernel Shells, Mater. Sci. Appl. 9, 2018, pp. 790–811 [1].
	doi:10.4236/msa.2018.910057.

**Value of the data**

• The data show the ultrastructure from SEM and TEM, giving access to nanometric sizes of the sample materials microstructure.
• Data could be highlighted for future studies related to potential applications [2], [3] of CKS/PKS as functional natural materials.
• The data could be highlighted for biomass elemental composition investigations.

## 1 Data

### 1.1 TEM micrographs

**Fig. 1:**
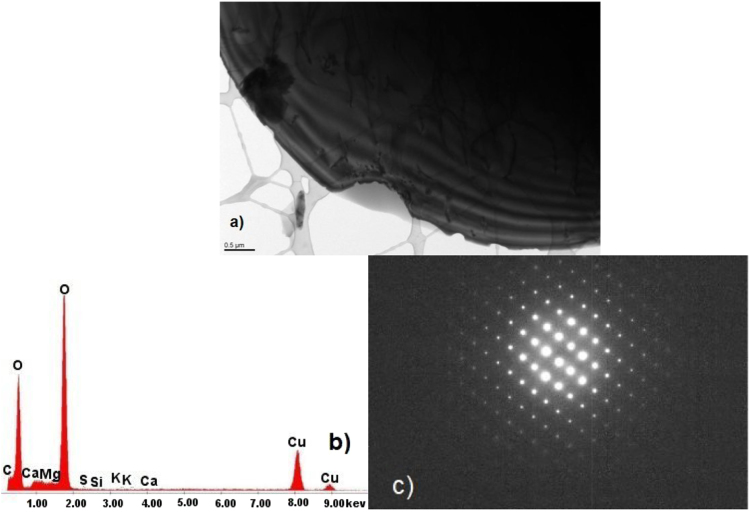
CKS Particle 1, a) 001 BF 4400×, b) EDS, c) EDP.

**Fig. 2:**
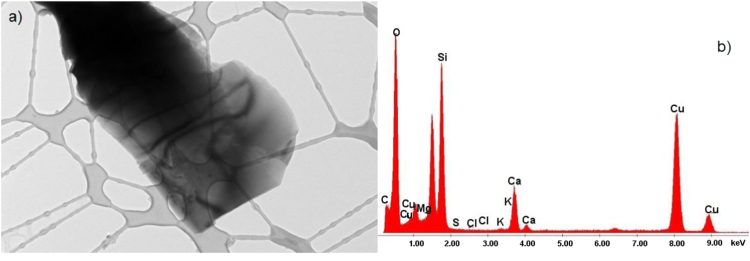
CKS Particle2, a) 003 BF 5600×, b) EDS.

**Fig. 3:**
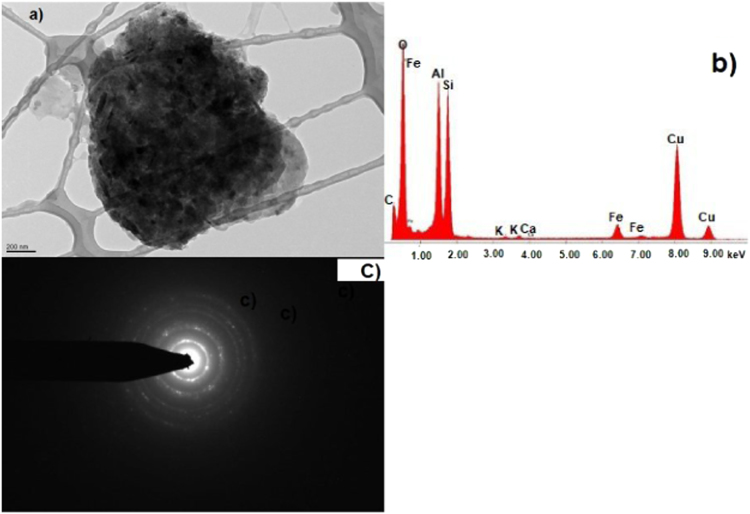
CKS Particle 3, a) 004 BF 10500×, b) EDS, c) Crystal structure not identified.

**Fig. 4:**
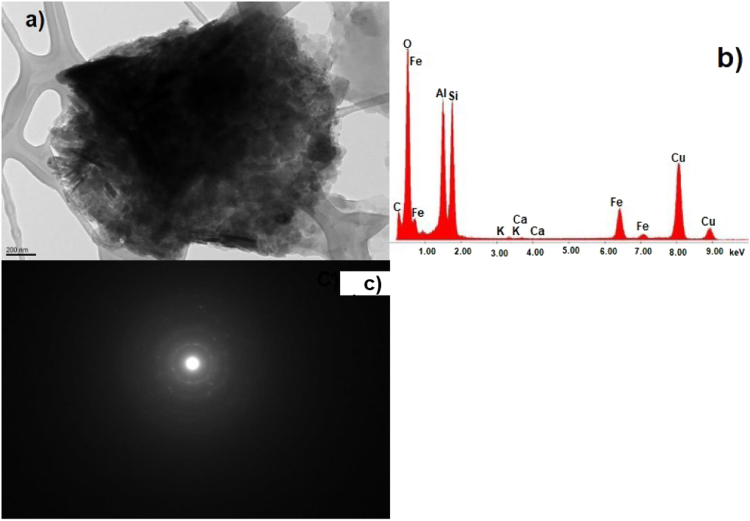
CKS Particle 4, a) 006 BF 10500×, b), c) EDP.

**Fig. 5:**
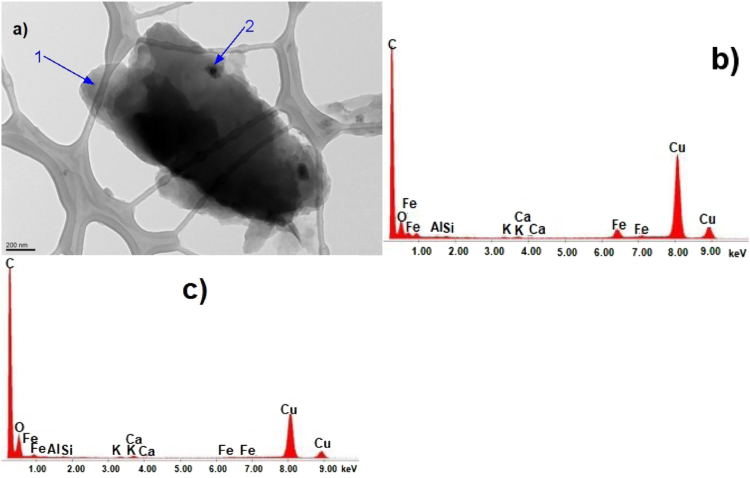
CKS Particle 5, a) 008 BF 10500, b) X-ray spectrum from region 1, c) X-ray spectrum from region 2 Fe-rich.

**Fig. 6:**
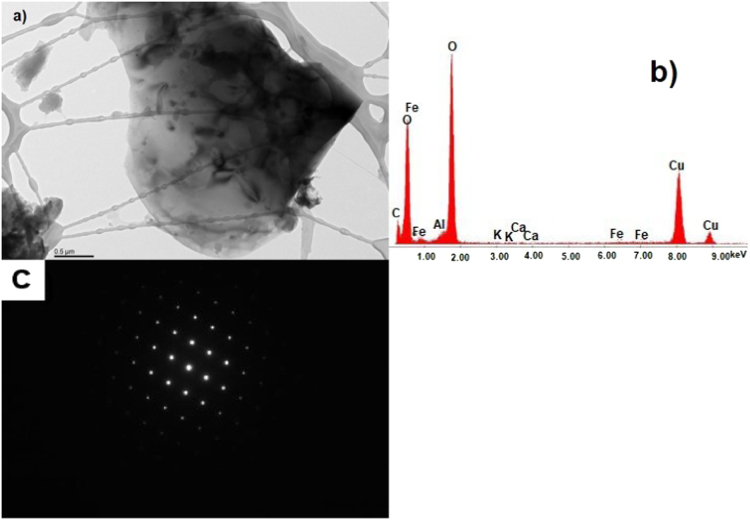
CKS Particle 6, a) 009 BF 5600 (crystalline), b) X-ray spectrum from particle 6 (silicon oxide), c) [11-1] zone axis EDP for quartz.

**Fig. 7:**
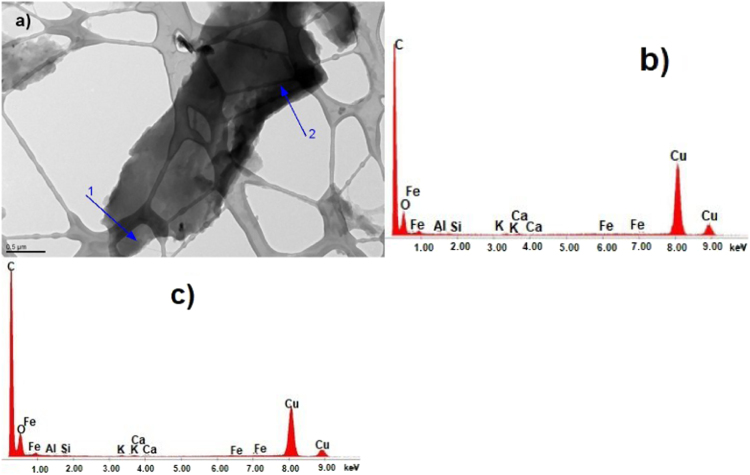
CKS Particle 7, a) 012 BF 5600× (noncrystalline), b) EDS spectrum from particle 7 region 1, c) EDS spectrum from particle 7 region 2.

**Fig. 8:**
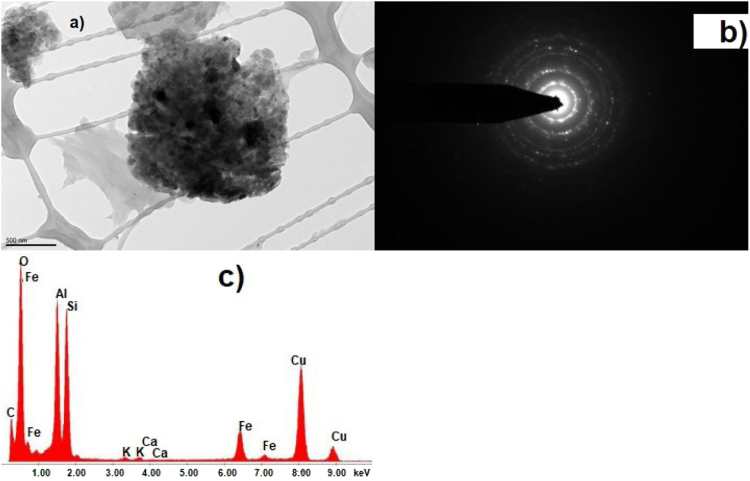
CKS Particle 8, a) 013 BF 7400×, b) SAEDP pattern (similar to particles 3 and 4), c) EDS micrograph.

**Fig. 9:**
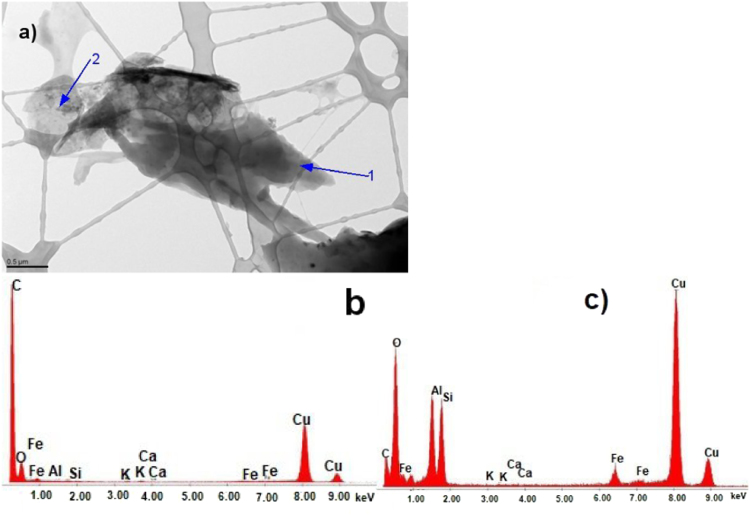
CKS Particle 9, a) 015 BF 5600 (arrows show location of EDS analyses), b) X-ray spectrum particle 9 region 1 (noncrystalline), c) X-ray spectrum particle 9 region 2 (crystalline).

The data show TEM micrographs of specimen particles of CKS. The data are Supplementary materials for a study on “Insight on the Ultrastructure, Physicochemical, Thermal Characteristics and Applications of the palm kernel shells material” [1]. Fig. 1 of Particle 1 shows typical morphology of a CKS particle. Fig. 2 of particle 2 shows a crystalline oxide particle. Fig. 3 of particle 3 shows a crystalline phase. Fig. 4 of particle 4 also represents a crystalline phase. In Fig. 5, particle 5 is mostly amorphous with some small crystalline regions; the locations of EDS analysis are shown with arrows. In Fig. 6, the particle is crystalline and EDS shows silicon oxide. Fig. 7 reveals a noncrystalline particle, also confirmed by X-ray spectra from both regions shown in Fig. 7b and c. Fig. 8a from particle 8 shows in Fig. 8b, a selected area electron diffraction pattern (SAEDP), and in Fig. 8c, an EDS graph. In Fig. 9 of particle 9, arrows show location of EDS analyses.

### 1.2 SEM micrographs

The micrographs included herein are voluntarily limited to TEM images of CKS for concision. Supplementary data related to SEM micrographs of PKS/CKS can be found at the above mentioned repository. In fact, particles of CKS show very clear plant cell/tissue structures. SEM/EDS data of plant cell/tissue of CKS showed a composition typically containing C, O and Silicon.

## 2 Experimental design, materials, and methods

### 2.1 CKS material

CKS waste was collected from local coconut commercial garbage zones at Missole. The shells were washed using a sodium hydroxide solution, rinsed by demineralised water and dried in an oven at 70 °C during 48 h, prior to analyses. The shells were also grounded and sieved. Powders obtained are from different sizes varying from 0.04 mm to 0.5 mm, and further weighted.

### 2.2 TEM/SEM specimen preparation

The prepared CKS powder was crushed with an agate mortar and pestle under distilled water to reduce the particle size. TEM specimens were made with the ground powder as follows: powder was sonicated for 5 min in distilled water then dropped onto a lacey carbon coated grid. A JEOL TEM, with a JSX-1000S Fluorescence Spectrometer X-ray analyser controlled by automated analysis software was used for the ultrastructure characterization. Low voltage was operated at relatively low electron accelerating voltage between 5 and 25 kV. This was particularly useful to increase image contrast and especially important for our biological samples [4].

For SEM observations, the CKS powder was spread onto an aluminum stub covered with a conductive carbon tape, such that the powder was evenly distributed on the surface of the carbon tape. It was also coated with a mixture of gold and palladium by a sputter coater (Polaron SC 7640). A JEOL JSM-6900 Low Vacuum SEM was used for the surface morphology observations. EDS for elemental analysis was performed by using the IMIX system.

## References

[1] Ntenga R., Mfoumou E., Béakou A., Tango M., Kamga J., Ahmed A. (2018). Insight on the ultrastructure, physicochemical and thermal characteristics and applications of the palm kernel shells. Mater. Sci. Appl..

[2] Liu M.Y.J., Chua C.P., Alengaram U.J., Jumaat M.Z. (2014). Utilization of palm oil fuel ash as binder in lightweight oil palm shell geopolymer concrete. Adv. Mater. Sci. Eng..

[3] Sumathi S., Bhatia S., Lee K.T., Mohamed A.R. (2009). Optimization of microporous palm shell activated carbon production for flue gas desulphurization: experimental and statistical studies. Bioresour. Technol..

[4] Khalil H.P.S.A., Yusra A.F.I., Bhat A.H., Jawaid M. (2010). Cell wall ultrastructure, anatomy, lignin distribution, and chemical composition of Malaysian cultivated kenaf fiber. Ind. Crop. Prod..

